# GC-CNNnet: Diagnosis of Alzheimer's Disease with PET Images Using Genetic and Convolutional Neural Network

**DOI:** 10.1155/2022/7413081

**Published:** 2022-08-09

**Authors:** Morteza Amini, Mir Mohsen Pedram, AliReza Moradi, Mahdieh Jamshidi, Mahshad Ouchani

**Affiliations:** ^1^Department of Cognitive Modeling, Institute for Cognitive Science Studies, Tehran, Iran; ^2^Department of Electrical and Computer Engineering, Faculty of Engineering, Kharazmi University, Tehran, Iran; ^3^Department of Clinical Psychology, Faculty of Psychology and Educational Science, Kharazmi University, Tehran, Iran; ^4^Department of Cognitive Psychology, Institute for Cognitive Science Studies, Tehran, Iran

## Abstract

There is a wide variety of effects of Alzheimer's disease (AD), a neurodegenerative disease that can lead to cognitive decline, deterioration of daily life, and behavioral and psychological changes. A polymorphism of the ApoE gene *ε* 4 is considered a genetic risk factor for Alzheimer's disease. The purpose of this paper is to demonstrate that single-nucleotide polymorphic markers (SNPs) have a causal relationship with quantitative PET imaging traits. Additionally, the classification of AD is based on the frequency of brain tissue variations in PET images using a combination of *k*-nearest-neighbor (KNN), support vector machine (SVM), linear discrimination analysis (LDA), and convolutional neural network (CNN) techniques. According to the results, the suggested SNPs appear to be associated with quantitative traits more strongly than the SNPs in the ApoE genes. Regarding the classification result, the highest accuracy is obtained by the CNN with 91.1%. These results indicate that the KNN and CNN methods are beneficial in diagnosing AD. Nevertheless, the LDA and SVM are demonstrated with a lower level of accuracy.

## 1. Introduction

AD is defined by irregular extracellular *β*-amyloid plaques and intraneuronal tau aggregation on a neuropathological level (neurofibrillary tangles). The concept of an AD continuum, which contains both typical and atypical manifestations of the disease, arose from observations that patients with various clinical appearances and progressions have identical neuropathological features [1, 2]. AD is a late-onset condition in more than 80% of patients (defined haphazardly as cases with 65 years or older). Mild cognitive impairment (MCI) is a dementia prodromal phase that affects the voice, visuospatial, praxis, and executive domains and worsens over time. On the other hand, patients with early-onset AD usually present with a more severe multidomain cognitive disorder impacting memory, concentration, vocabulary, visuospatial, and executive functions at the time of diagnosis. In patients with early-onset AD, except for the elderly, MCI rarely precedes primary cognitive dysfunction, which also develops more rapidly to severe steps. As a result, in 2010, 2011 [3, 4], and 2014, atypical AD variations were applied to the updated diagnosis guidelines for AD. The most recent updated form [5] involves (1) a clinical phenotype associated with one of the atypical forms of dominant, progressive, frontal and (6) logopenic variant, visual/posterior variant and (2) biochemical, genetic, and/or in vivo molecular imaging symptoms confirming AD diagnosis. Nevertheless, new clinical phenotypes of AD have been recorded recently in patients with semantic variant predominantly progressive aphasia [6] or corticobasal syndrome [7]. These innovative clinical variations add to the taxonomy of AD, accentuate a broad range of patient features. Most notably, the diagnosis of early-onset variants of Alzheimer's poses critical challenges [8] underlines the significance of biomarkers for detection in vivo.

FDG-PET is a promising modality for forecasting adaptive brain alterations in AD, detecting variations early in the disease, and recognizing AD from other dementias. Several studies on the efficacy of FDG-PET in AD have been published over the last three decades. A meta-analysis of 27 FDG-PET investigations in the diagnosis of AD finds a 91% (95% confidence interval, 86%–94%) and 86% (95% CI, 79%–91%) pooled sensitivity, and 86% (95% CI). The study included 119 papers examining the function of different diagnostic methods in AD. The meta-analyzes find that FDG-PET has outstanding diagnostic accuracy [9, 10] compared to other diagnostic approaches such as clinical guidance, MRI, CT, SPECT, and biomarkers. Besides, tests have shown that FDG-PET can differentiate patients with AD from stable controls and dementia from other diseases. The FDG-PET is to recognize 98 and 99 percent AD patients with normal SN and SP subjects, 99 and 98 percent DLB with SN and SP patients with 99% and 71 percent SN and SP and 99 and 65 percent FTD patients with SN and SP, according to Mosconi et al. [11]. Neuroimaging has been critical in supporting underlying pathophysiological hypotheses regarding the condition over the past two decades, and it has primarily been linked to the evolution of diagnostic methods. The conditions for amnestic (typical) AD causes have been revised: MRI hippocampal atrophy, temporoparietal hypometabolism of FDG-PET, and elevated fibrillar amyloid PET-amyloid accumulation in the brain. In particular, exposure to imaging biomarkers raises the likelihood of an AD diagnosis even under preclinical/predementia circumstances [5].

Is molecular PET imagery able to explain the phenotypic variety of AD and explain whether and how pathologic *β*-amyloid and tau proteins show the clinical appearance of the disease? To date, amyloid PET tests have seen diffuse cortical *β*-amyloid deposits in patients with average or atypical early-onset AD, irrespective of clinical presentation. The relationship between cognitive profile, metabolic transition, and irregular protein distribution has been small. Furthermore, this radiotracer family has not shown a distinct geographic trend between focal and diffuse AD [12, 13]. On the other hand, PET tracers that target tau have revealed a close link between tau deposit distribution and clinical phenotype.

For the purposes of monitoring the progression of AD, we examined genes that have significant correlations with statistical properties of three PET tracers other than the ApoE genotype. In this article, 37 characteristics are discussed to assist in diagnosing Alzheimer's disease. PET images provide inputs for different parts of the brain depending on their frequency dependence. Nearest-neighbor (KNN), support vector machine (SVM), linear discrimination analysis (LDA), and convolutional neural network (CNN) are four machine learning approaches used to diagnose Alzheimer's disease. Several reduced features are used to create the input layer, while two MCI labels and the normal value are used to construct the output layer.

The following sections of the present paper outline: Section 1 describes medical imaging and key medical imaging characteristics and quality factors.  Section 2 reviews many relevant papers in medical image processing and studies some image processing methods for improving medical images that researchers have proposed in their papers.  Section 3 is the core of the present research paper. This section explains some of the significant engineering subjects related to image processing, general, and medical imaging, particularly in Section 4. The evaluation metrics are discussed in Section 5. Finally, Section 6 summarized the numerical results and future works.

## 2. Literature Review

Alzheimer's disease is a neurodegenerative disease with distinct pathologic characteristics. Although cortical and hippocampal neuronal dysfunction and generalized gray matter atrophy are hallmarks of Alzheimer's disease, patients can also experience gradual disconnection of cortical and subcortical regions attributable to white matter injury. AD is a progressive disease that worsens over time. The ApoE genotype *ε*4 is well known as a genetic risk factor for AD. Furthermore, PET/MRI is a systematic instrument for clinical detection of AD by identifying changes in the brain. We looked at single-nucleotide polymorphisms (SNPs) focused on whole-genome sequencing (WGS) data in this research.

The biochemical structures found with the gold standard of PET imaging of fluorodeoxyglucose (FDG) strongly mimic the cortical distribution of tau protein: hypometabolism is a pathologically intimate result of tau deposition [13, 14]. In short, in the early-onset Alzheimer varieties, the function and density of tau aggregation are locally linked to cognitive effects, cerebral blood pressure, atrophy, and metabolic changes, while *β*-amyloid is diffusing [13]. The area of study in brain imaging genetics explores the effect of genetic variations on brain imaging phenotypes. It examines how genetic variations such as single-nucleotide polymorphisms (SNPs) and quantitative traits (QTs) derived from brain imaging evidence contribute to phenotypical features and molecular mechanics in complicated brain conditions. Single voxels [14] or regions of interest (ROIs) [15–17] in the brain are used to calculate imaging QTs. An ROI is a predetermined brain region consisting of an anatomical/functionally annotation similar cluster of voxels. The ROI number (ten hundred) in the cortex is significantly smaller than the voxel number (tens of thousands to many millions).

Recent advancements in obtaining multimodal neuroimaging technology inherently have precise voxel-level knowledge, which opens up a plethora of possibilities for investigating fine-grained brain anomalies. Voxelwise methods to investigate genetic implications for voxel-based brain measures have been suggested in brain imaging genetics. Stein et al. [14] suggested that GWAS (vGWAS) could be included in an AD analysis to evaluate relationships paired by 448,293 SNPs and 31,622 voxels. Hibar et al. [18] proposed the voxelwise gene-wide interaction study (vGeneWAS), which compared the combined influence of several SNPs within a gene to voxel-level measures using a multivariate model. In their study, He et al. [19] studied several methods for selecting data features to achieve dimensionality reduction. Chen et al. [20] developed DeepM6ASeq-EL, which utilizes an ensemble of five LSTMs and CNNs with a hard voting strategy. According to Xu et al. [21], pathogenesis can be represented using a directed graph (PN) in a heuristic way.

When vGWAS (e.g., CSMD2 and CADPS2) and vGeneWAS (e.g., GAB2) have detected specific genes, no primary genetic imaging links have been found have survived the correction of several tests. In organized sparse learning, Du et al. [40] proposed two new penalties to strengthen the fused lasso and the graph/network-driven lasso penalties. They penalized the SCCA model in both ways and proposed an optimization algorithm to solve it. The suggested SCCA approach had a clear upper limit on grouping results positively and negatively correlated variables. In discovering biologically significant imaging genetic associations, the suggested technique detected higher canonical correlation coefficients and captured simpler canonical weight patterns. Auditory verbal learning test delayed recall (AVLT-DR) regressing 6-month AVLT-DR (AD neuroimaging Initiative database) scores in 394 individuals with adequate knowledge at baseline AVLT-DR scores. According to the findings, loss of practice effect over six months can be as effective as biomarkers in predicting 6-year AD risk.

The study by Yao et al. [41] proposed voxel-wise enrichment analysis that integrates brain-anatomic annotation results as an efficient and robust means for mining regionally based imaging genetic associations recognizing the mutual impact of weak voxel-level signals. In order to investigate the genetic effects of imaging on the brain, the proposed technique has demonstrated to be both scalable and effective. In a study by Zhao et al. [42], the Multiple Kernel-based Fuzzy SVM Model with Support Vector Data Description (MK-FSVM-SVDD) was proposed in order to predict DBPs. In Yan et al. [43], plasma-activated water (PAW) and heat-moisture treatments (HMT) were combined to study the structure, physical properties, and in vitro digestibility of waxy (WMS) and normal maize starches (NMS). In Shi et al. [44], the effect of WSG and its impact on steamed bread quality were studied. Increasing ultrasonic intensity first increased and then decreased the complex index (CI) of the WSG. Nejatishahidin et al. [45] developed a novel pose estimation model for object categories that can be effectively applied to previously unknown environments. Eslami et al. [46] showed that attention-based multiscale convolutional neural networks (A+MCNNs) could improve the automated detection of common distress and nondistress objects in pavement images. In this study, Dubois et al. [47] investigated epigenetic processes as they relate to psychiatric disorders and traumatic or stressful events, family relationships, and also gut microbiota. Wang et al. [48] used the BP neural network algorithm to train the input value of the network marketing and to judge the risk. Prasad et al. [49] used response surface methodology (RSM) and artificial neural network (ANN) to predict the color removal by adsorption. Rezaei et al. [50] introduced a data-driven method to segment hand parts from depth maps without requiring any additional effort to obtain segmentation labels. In their study, Chandra et al. [51] examined in vivo molecular imaging in relation to amyloid, tau, and microglial activation in AD pathology. As part of the study, PET imaging tests were examined as possible biomarkers and ways to control disease development (see Table 1). In recent research, metaheuristic optimization methods have grown more attractive [52, 53]. Because they can solve multiple-objective solutions and nonlinear formulations, metaheuristics are increasingly being utilized to find high-quality solutions to a growing number of complex real-world problems [54–58]. Optimization approaches underpin a wide range of essential tasks, and they may be used to solve a wide range of image segmentation issues in medicine [59–63]. In summary, imaging genetics investigation focuses on ROI-level phenotypes such as (i) low dimensionality relative to voxel-based computational strength approaches and (ii) structural or functional ROI annotations to indefinite analysis. AD is a progressive disease that worsens over time. The ApoE genotype *ε*4 is well known as a genetic risk factor for AD. Furthermore, PET/MRI is a systematic instrument for clinical detection of AD by identifying changes in the brain. We looked at single-nucleotide polymorphisms (SNPs) focused on whole-genome sequencing (WGS) data in this research. We discovered several SNPs that have a strong link to PET imaging quantitative traits (QTs). Moreover, the classification is done to diagnose AD based on the frequency of different brain parts in PET images. Analysis metrics are used to illustrate the results. Machine learning is also widely used in biological applications, such as optimization [63, 64], feature extraction [65, 66], and diagnosis of tumors [67]. The applications of deep learning method are infection disease detection [68], economical application [69], cancer research [70], brain tumor detection [71, 72], fatigue detection [73], environmental science [74], federated learning [75], facial expression detection [76], and healthcare analysis [77]. Moreover, some metaheuristic methods are aquila optimization [78], reptile search method [79], genetic algorithm [74], and so on [80].

## 3. Methods and Materials

### 3.1. PET Imaging Genetics

PET imaging genetic expression can be precisely accomplished by radiolabeling samples that only bind certain parts of the target molecule (e.g., protein, mRNA, or DNA) or radiolabeling samples, which are explicitly metabolized by a particular enzyme or sequence of reactions leading to a radiolabeling complex that is “trapped” in the tissue. There are also instances of the nuclear medicine direct imaging model. In various areas, including neuroscience studies, PET imagery of receptor density/occupancy with little radio-labeled molecular sensors is widely used. Another instance of direct molecular imaging that has progressed over the last 30 years [81] is picking cell surface-specific antigens or epitopes with radiation-labeled antibodies.

### 3.2. Convolutional Neural Network

A CNN is a deep learning (DL) method that can take an input matrix and assign importance (learnable weights and biases) to different aspects/objects while also distinguishing between them. In comparison to other classification methods, a CNN requires significantly less preprocessing. In spite of the rudimentary design of filters, CNN can learn these filters/characteristics with enough training. CNN architecture was inspired by the structure of the visual cortex, which is similar to the pattern of communication between neurons in the human brain. Individual neurons are only capable of responding to stimuli that are located within the receptive field, a small portion of the visual field. When multiple such fields collide, the entire visual field becomes occupied [82]. Manual attribute extraction methods, including such texture analysis, are used in the majority of recent radionics experiments, accompanied by traditional machine learning (ML) methods, like random forests and support vector machines (SVM) [83]. There are a few distinctions to be made between those approaches and CNN. To begin with, CNN does not necessitate feature extraction by hand. Second, human experts are rarely used to segment tumors or organs in CNN architectures. Third, because millions of learning parameters are necessary to predict, CNN is much more data hungry and computer intensive, and GPUs are required for model training. Among the building blocks of CNN architecture are convolution layers, pooling layers, and fully connected layers. One or two fully connected layers follow a stack of multiple convolution layers and a pooling layer in the typical architecture. The way the input data is converted to output data that uses these layers is called forward propagation. Though 2D-CNN is used for convolution and pooling, the associated three-dimensional (3D)-CNN operations can also be applied [84].

### 3.3. Support Vector Machine (SVM)

SVM is the most used (ML)-based pattern classification technique today. It was created by Vapnik in 1995 and is centered on mathematical learning theory. The main goal of this methodology is to use various types of kernel functions to project nonlinearly separable samples onto a higher dimensional space. Kernel methods have gotten much attention recently, thanks to the growing success of SVM [85]. Kernel functions are essential in SVM for bridging the gap between linearity and nonlinearity. The least-square SVM technique is another helpful SVM methodology for classification tasks. For grouping, extreme learning machines, fuzzy SVMs, and genetic algorithm-tuned expert models can all be used. Three different kernel functions, namely linear, polynomial, and RBF kernels, were tested in this analytical work [85].

### 3.4. K-Nearest-Neighbor Classifier (KNN)

The KNN classifier is a common and useful data mining tool. KNN classifies each test sample based on its *k* nearest neighbors. The distance between the research samples and all training samples should be determined to locate the *k* nearest neighbors. It necessitates a significant amount of computing overhead in the case of big data. To discover the *k* nearest neighbors total training sets, some researchers use distributed frameworks like Hadoop [86]. These methods usually yield the same *k* nearest neighbors but at the expense of a massively distributed system. On the other hand, other authors consider searching for the closest neighbors in a smaller training data set. Using a KNN classifier on big data necessitates many computing resources. The class mark of a test sample is calculated using the *k* closest samples from the training data set in this classification process. The distance between the research samples and all training samples should be measured to locate the *k* closest neighbors [86].

### 3.5. Linear Discriminant Analysis (LDA)

Fisher's linear discriminant is a statistical and another tool for evaluating a linear mixture of features that describes or distinguishes two or more types of objects or events. Fisher's linear discriminant is a generalization of LDA. The resulting combination may be utilized as a linear classifier or, more broadly, as a dimensionality reducer before additional classification. Discriminant analysis is employed where categories are known a priori (unlike in cluster analysis). Each scenario requires a score on one or more quantitative predictor variables and a score on a group indicator [87]. In its most abstract form, discriminant function analysis involves grouping, classifying, or categorizing objects into related groups, classes, or categories.

### 3.6. Performance Metrics

**Figure 1:**
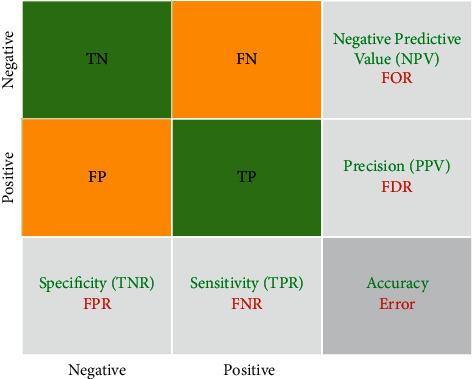
The confusion matrix.

Patients are assigned to one of the four cells identified as *d* in Figure 1 according to classification outcomes and regardless of whether or not the target diagnosis is focused on the classification result and whether this evaluation has produced either a positive outcome (the individual seems to be the person) or a negative outcome (the person does not seem to have the condition) (the person seems not to have the condition). The numbers of individuals in each of the four cells will then be employed to calculate sensitivity, specificity, and predictive values, which are based on the following formulas [88] as expressed as percentages:

(1) $$\begin{array}{c} \text{Sensitivity}=\left[\frac{TP}{TP+FN}\right]\times 100,\\ \text{Specificity}=\left[\frac{TN}{TN+FP}\right]\times 100,\\ \text{Positive predictive value}\left(PPV\right)=\left[\frac{TP}{TP+FP}\right]\times 100,\\ \text{Negative predictive value}\left(NPV\right)=\left[\frac{TN}{TN+FN}\right]\times 100,\\ \text{Accuracy}\left(ACC\right)=\left[\frac{TP+TN}{TP+TN+FP+FN}\right]\times 100. \end{array}$$

These are the criteria cited by researchers and clinicians related to sensitivity, specificity, and predictive values to determine the impact of a classifier outcome—i.e., often as percentages but usually as decimal fractions, preferably with an acceptable confidence interval of 95 percent. The simplicity, and even familiarity, of these four metrics, on the other hand, can obscure the existence of several complications that are often ignored. There could be flaws in either the comparison standard or the exam or both. The four metrics cannot be considered indisputable and unchangeable test characteristics: the rigor of the evaluation and the occurrence of the target condition in the study determine the measurements inserted into the cells of Figure 1 [88].

## 4. Results and Discussion

### 4.1. Data Collection

For this paper, the information was collected for the ADNI data set. The ADNI was founded by Principal Investigator Michael W. Weiner, MD, in 2003 as a public-private study. The primary objective of ADNI was to determine whether it was possible to track the progression of MCI and early AD with serial RMI, PET, and other biological markers, as well as clinical and neuropsychological evaluations. Several of the participants were able to obtain baseline and follow-up measurements of FDG. During the study trials, PET scans with 18F-AV45 as well as 11C-PiB were conducted for imaging of amyloid plaques. For each baseline and study, structural MRIs (1.5T or 3T, magnetization prepared rapid acquisition gradient echo) are obtained. The ADNI database also included Apolipoprotein *E* (APOE) genotypes, CSF scales, and clinical evaluations.

### 4.2. Descriptive Statistics

**Figure 2:**
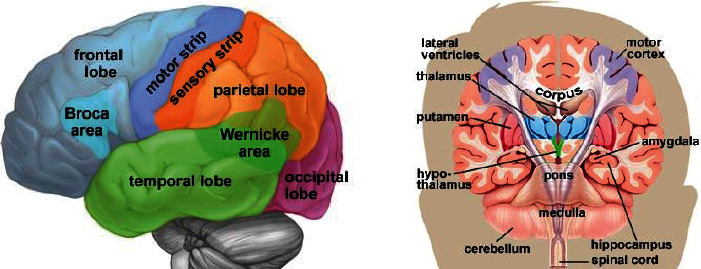
The anatomy of the cerebrum in the human brain [89].

In this study, 75 topics were selected from the ADNI GWAS data set [89] with more than seven years of FDG PET, structural MRI, [18F] AV45, and [11C] PIB scans. All PET images accompanied by structural MRIs were imported into the ADNI database. The regions of interest (ROI) in a high-resolution MRI prototype were drawn manually. The variables used are presented in Table 2. The orbital cortical, prefrontal, superior frontal, lateral temporal, parietal, medial precuneus, occipital, anterior cingulate, and posterior cingulate make up the global cortex. The ROI of gray matter in the cerebellum is utilized as target tissue, and the 34 ROIs in the normal MNI space that consists of cerebellum were used as template ROIs for all subjects. Refer to Figure 2 for an illustration of the brain's configuration.

**Figure 3:**
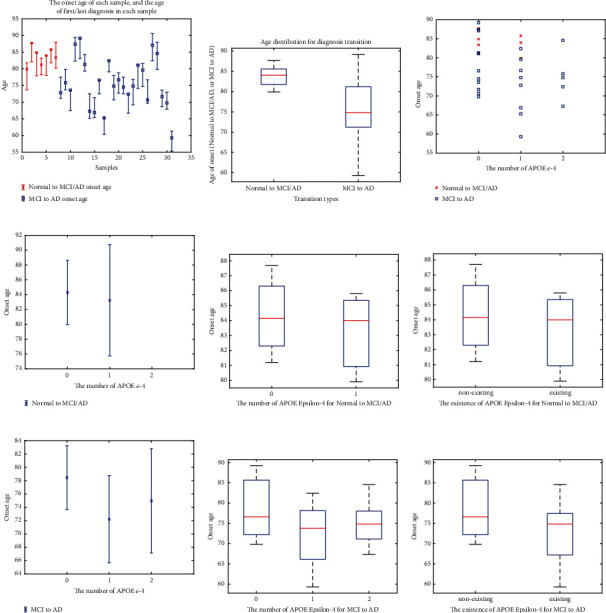
Results of descriptive statistics.

The comparative study of the patients with Alzheimer's disease is seen in Figure 3. Longitudinal Alzheimer's research is critical because the abnormality and order of shifts with each biomarker vary dramatically as Alzheimer's progresses over time (see Figures 3(a) and 3(b)). The quantitative PET approach is regarded as a crucial method for tracking and assessing Alzheimer's disease development. Standardization and optimum use of PET in AD imaging include evaluating single or multiparametric PET output in the evaluation of patients. Based on the analysis of 32 patients, some of them changed the progression and Alzheimer's between normal to MCI of Alzheimer and MCI to AD. Based on the results in Figure 3(b), people with new symptoms of AD and MCI is detected in almost 85 years old. In other words, this group saw the first effects of AD on their brains (age between [80 and 86]). However, the progression from MCI to AD is revealed for a wide range of ages [60–90] years with a mean of 75.

**Figure 4:**
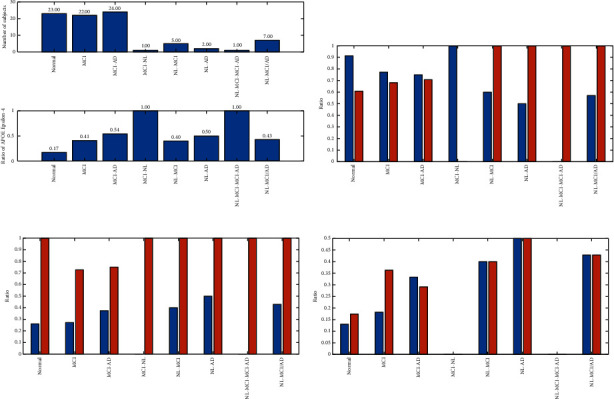
Results of diagnosis using statistical analysis.

Regarding Figure 3, in the normal group, there was no discrepancy among converters and nonconverters in age, APOE carriers. It also exists in APOE-*ϵ* − 4 for decreasing the onset age of AD. That is why the age range of people with APOE-*ϵ* − 4 is lower than people without this carrier (see Figure 3(f)). It has occurred for people's AD progression. The progression of MCI to AD for people without APOE-*ϵ* − 4 has occurred for people for age between [70 and 90] (see Figure 4).

### 4.3. Results of Diagnosis Using Statistical Analysis

In this paper, three SNPs are used for the diagnosis of AD in the sample patients. The essential SNP is shown in Table 3.

**Figure 5:**
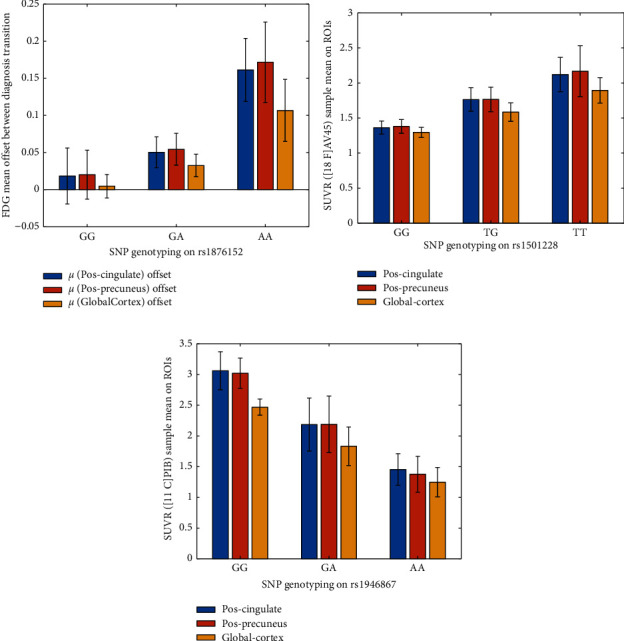
Standard uptake values ratios for three essential SNP.

The rs1876152 SNP has three variations in the sample domain: GG, GA, and AA. Three participants were selected to display their standard uptake values ratios in cortexes such as the pos-cingulate and pos-precuneus, frontal, parietal, and occipital. The presented findings support the hypothesis seen in Figure 5 that the detected SNP can substantially affect the decreasing pace of FDG uptake. The ApoE genotypes of the participants are all the same, which is *ε*4 and *ε*3. As a result, the various declining speeds are unrelated to the ApoE difference in this situation. According to the findings, the suggested SNPs have a more significant association with QTs than the SNP from the ApoE gene. The genotypes rs1876152 on chromosome 5, rs1501228 on chromosome 1, and rs1946867 on chromosome 4 all have a strong linear association with FDG, [18F]AV45, and [11C]PIB measurements, respectively. FDG, [18F]AV45, and [11C]PIB PET measurements all show a strong association with the genotypes rs1876152, rs1501228, and rs1946867, respectively. The ApoE genotype is a coarser genetic risk factor for AD. To better track the progression of AD, our research identified genes that have strong associations with quantitative characteristics of three PET tracers other than the ApoE genotype. The current ADNI research will observe the assessment of the three genotypes in controlling AD development.

The *Y*-axis in Figure 5 indicates the average discrepancy in FDG measurements before and after the diagnosis process for seven years. The error bar represents the 95% confidence interval for the discrepancy in means. On the *X*-axis, the SNP genotype rs1876152 has three variations: GG, GA, and AA. The SNP genotype rs1501228 has three variants: GG, TG, and TT, while rs1946867 has three variants: GG, GA, and AA. The subjects with GG alleles have a minor difference in FDG measurements between two transformations (see Figure 5(a)). After the transition, the FDG SUVR decreases the most when the gap in AA alleles is more significant.

### 4.4. Results of Diagnosis Using Machine Learning

**Figure 6:**
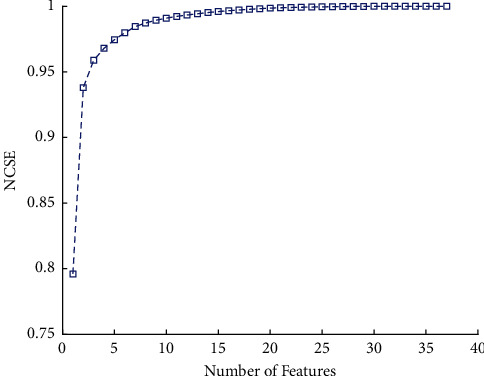
Normalized cumulative summation of sorted eigenvalues for feature reduction.

In this paper, 37 features are used for the diagnosis of AD in patients. The input features are indicated in Table 2 that are different parts of the brain PET frequency based on PET images. For the implementation of machine learning methods, the main features should be normalized. The normalizations have been done in each variable to range data between −1 and 1. The next step is to decrease the number of variables. In this part, for reducing the feature principle component analysis of use. The normalized cumulative summation of sorted eigenvalues (NCSE) is illustrated in Figure 6. Based on the results of feature reduction, the first ten features have a 99% power of all 37 inputs.

**Figure 7:**
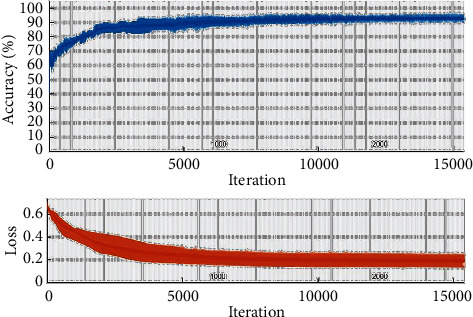
The training process of the CNN method.

In this paper, four machine learning methods consist of KNN, SVM, LDA, and CNN to diagnose AD. Ten reduced features are used as input layers, and two labels of MCI and normal value are used as output layers. The results of classification are reported for 511 patients and 311 normal people. The performance metrics are illustrated as confusion matrix and ROC curve. For CNN methods, the training process is shown in Figure 7.

Pseudo-code of the presented procedure.

  Collecting PET ()
 
**For All** (images)
  Feature extraction ()
 
**End for ***A* = Extract (Covariance Matrix)
  Calculate (Eigen Vector (A))
  Feature reduction ()
  Classification ()
  Performance Analysis ()

**Figure 8:**
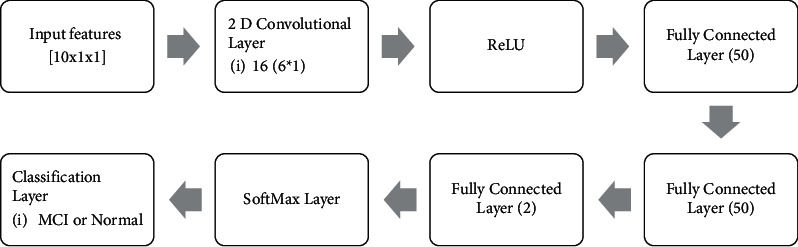
The architecture of the CNN method.

**Figure 9:**
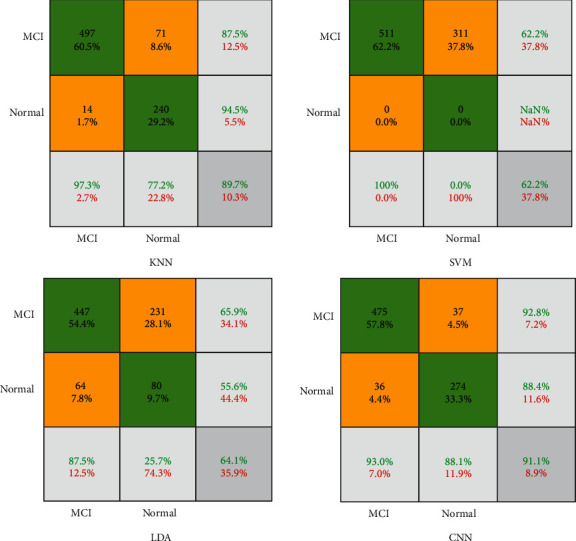
The confusion matrix of the presented classifiers.

**Figure 10:**
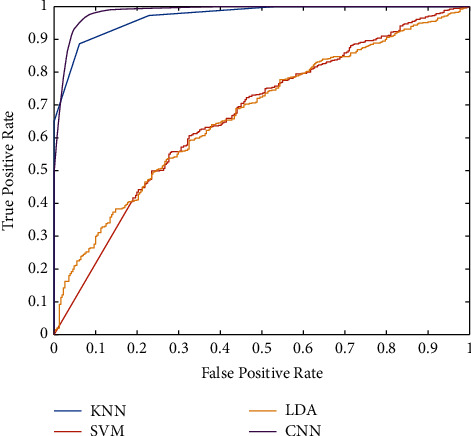
The ROC curve for the utilized machine learning classifiers.

Regarding Figure 7, the process is repeated until the accuracy and loss value are stable. Additionally, a convolutional layer and activation layer are employed for classification, as shown in Figure 8. The ReLU activation function is the best choice for CNN techniques for removing negative values. Three fully connected layers with 50, 50, and 2 are used for changing data size to two categories. Finally, the SoftMax layer connects the architecture to the output layer. Results are indicated in the form of Figures 9 and 10.

Based on the confusion matrix, the green cells are true or correct in diagnosis versus orange cells as false diagnosis value. Regarding the results of the KNN method in Figure 9, from 511 MCI samples, 497(97.3%) of them are detected successfully. In other words, the sensitivity of the KNN method is 97.3% for the diagnosis of MCI. On the other hand, from 311 normal samples, KNN finds 240(77.2%) of them correctly. This parameter is also called specificity. Based on the results of KNN, the precision of the method is 87.5%. It means that 497 + 71 persons are detected as MCI patients and that 87.5% of them are correct. Finally, the accuracy of the KNN is 89.7%. For a description of the SVM method, the method could not detect any types of patients and the accuracy are 62.2%. Nevertheless, the sensitivity of the SVM is 100%, and the specificity is zero. It means that none of the normal people is detected. About the LDA method, the sensitivity is 87.5%, while the specificity is 25.7%. It means that only 80(25.7%) normal persons are detected successfully. In this paper, we presented a CNN architecture to find an accurate model for AD diagnosis. Based on the results of the CNN method, the sensitivity is 93%. The CNN method could diagnose 88.1% of normal persons. Regarding the results of the classification, the highest accuracy is belonging to CNN with 91.1%. The ROC curve is depicted in Figure 10 for a good description of the classifiers. Regarding Figure 10, the *x*-axis is the false-positive rate (FPR), and the *y*-axis is the true-positive rate (TPR). The method with lower FPR and higher TPR is desirable. Results show that the KNN and CNN method is a desirable method for diagnosing AD. However, the LDA and SVM are illustrated with lower accuracy.

## 5. Discussion

Clinically, image processing employing a CNN has gained considerable attention as a form of artificial intelligence. Its high performance in image recognition makes CNN a branch of deep neural networks (so-called deep learning) that is recognized to be highly useful for image analysis. A recent study employed a CNN to automatically diagnose tuberculosis from chest radiographs. Through the use of a CNN, we were also able to segment brain tumors and predict genotype from magnetic resonance images. One study found that dynamic contrast agent-enhanced computed tomography was very effective in distinguishing liver masses. PET/CT imaging has also been successfully used with CNN algorithms. In more recent years, generative adversarial networks (GANs) have been used to increase super-resolution efficiency, yet these approaches have been limited by the difficulty of training GANs, which is notoriously difficult. While deep neural networks have been effectively used for PET image denoising and radiation dose reduction in a number of recent articles, the application of deep learning for PET imaging is a less-explored research domain. The super-resolution issue, unlike the denoising problem, tries to build a clearer image from a hazy one while preferably maintaining edges. As a consequence, super-resolution requires different network architectures and data preparation procedures than denoising. The future work on super-resolution PET will utilize a diverse range of techniques, including both (classical) penalized deconvolution using joint entropy and deep learning using CNN. For future work, it is better to use some powerful feature extraction methods to select the more reliable features for diagnosis with PET images.

## 6. Conclusion

We examined genes that were significantly correlated with statistical properties of three PET tracers that are not associated with ApoE genotype for the purpose of monitoring AD progression. This article discusses 37 characteristics relevant to the diagnosis of Alzheimer's disease. A PET image provides inputs for different parts of the brain depending on their frequency dependence. We discovered several SNPs that have a link to PET imaging quantitative traits (QTs). Moreover, the classification is done to diagnose AD based on the frequency of different brain parts in PET images. The results are illustrated with performance analysis metrics. According to a study of patients, some improved their Alzheimer's development from mild to MCI and MCI to AD. According to the findings, individuals as young as 85 years old have additional signs of Alzheimer's disease and MCI. In other words, this population saw the earliest signs of Alzheimer's disease in their brains (age between [80 and 86]). The progression from MCI to AD, on the other hand, is visible for a wide variety of ages [60–90] years, with a mean of 75. The genotypes rs1876152, rs1501228, and rs1946867, respectively, have a clear linear relationship with FDG, [18F] AV45, and [11C] PIB scales, according to the findings. According to the results, the proposed SNPs have a stronger connection to QTs than the SNP from the ApoE gene. Our study examined genes that have significant correlations with statistical properties of three PET tracers other than the ApoE genotype in order to help monitor the evolution of AD. In this article, 37 characteristics are used to diagnose Alzheimer's disease in patients. Different areas of the brain frequency dependent on PET images are used as input functions. According to the effects of feature reduction, the first ten functions have a 99 percent impact on all 37 inputs. KNN, SVM, LDA, and CNN are four machine learning approaches used to diagnose Alzheimer's disease. The input layer consists of ten reduced features, while the output layer consists of two MCI labels and the normal value. According to the findings of the KNN process, 497 (or 97.3 percent) of the 511 MCI samples were successfully detected. In other words, the KNN system has a sensitivity of 97.3 percent for diagnosing MCI. KNN, on the other hand, accurately identifies 240 (or 77.2 percent) of 311 standard samples. The SVM system failed to detect any of the patients, with a 62.2 percent accuracy. Despite this, the SVM's sensitivity is 100 percent, and its specificity is nil. CNN has the best accuracy rate of 91.1 percent when it comes to classification data. The findings suggest that the KNN and CNN methods are suitable for diagnosing Alzheimer's disease. The LDA and SVM, on the other hand, are depicted with less precision.

## Figures and Tables

**Table 1:** The literature reviews.

Ref	Probe	Results
[22]	[^11^C]PBB3	[^11^C] PBB3 was substantially higher in Alzheimer's disease than controls in medial temporal areas, including the hippocampus

[23]	[^11^C]PBB3	In neocortical regions, particularly the medial temporal-co, significant variations in tracer uptake were found, while the Alzheimer's disease spectrum was comparable to normal controls. The group also experienced MRI medial time atrophy. Besides, the intake of cognitive status in front and temporoparietal joints, limbic, paralimbic, and frontoparietal zones, was positively linked with dementia, and frontal uptake of Alzheimer's patients in frontal regions was also correlated positively with frontal executive dysfunction

[24]	[^11^C]PBB3	[^11^C]THK5351 displayed larger percept in the temporal lobe of the medium and lateral lobe, and the reverse was shown in a combination of patients of Alzheimer's disease and mild cognitive impairment. [11C]PBB3 is implicated in the uptake of PET amyloid. The brain uptake of [^11^C]THK5351 and [^11^C]PBB3 has shown to be adversely linked to cognitive efficiency
	[^11^C]THK5351	

[25]	[18F]THK5317	The lat-temporal, lat-occipital-, inf-parietal, anterior, lat-occipital-co, and precuneus patients with mild cognitive impairment and Alzheimer's disease have greater tau connection than in healthy individuals. In PET, tau retention and fluorodeoxyglucose uptake were harmful in the frontal-Co, but the tau and the amyloid bonding were positive in the neocortex

[26]	[^18^F]THK5351	As contrasted to healthy controls, the eroded WM, fusiform gyrus, inf-temporal-co, lingual gyrus, mid-temporal gyrus, occipital-Co, parietal-Co, post-cingulate, and precuneus all indicated increment tracer absorption

[27]	[^18^F]THK5317	The occipital regions, the mid-frontal and post-cingulate gyri, the parietal operculum, the precuneus, and the parahippocampal, fusiform, intermediate, lower, and superior temporal gyri, were observed to be adversely linked to memory in Alzheimer's patients. Fluorodeoxyglucose-PET studies, which revealed an essential correlation between tau binding and cognition, affected the impact of in vivo tau binding on cognition

[28]	[^18^F]THK5351	Uptake of [^18^F]THK5351 was greater in Alzheimer's patients in the cerebral temporal and occipital regions than in healthy controls; in the hippocampus, [^18^F]AV1451 uptake was higher
	[^18^F]AV‐1451	

[29]	[^18^F]AV‐1451	In all four lobes of the cortex as well as of the hippocampus, the connections with Alzheimer's disease were more robust in comparison with stable controls

[30]	[^18^F]AV‐1451	In Alzheimer's disease patients in hippocampal and extensive cortical areas, tracer retention was more remarkable compared to control

[31]	[^18^F]AV‐1451	A significant proportion of cortical regions examined in Alzheimer's disease have greater tau uptake than controls. This condition persisted in mild cognitive impairment for the entorhinal-Co

[33]	[^18^F]AV‐1451	The cortical preservation of [^18^F]AV1451 was higher than the controls for the temporoparietal, parietooccipital, precuneus post-cingulate, and frontal areas in mixed patient groups. In the entorhinal, parahippocampal, inferior temporal, and fusiform-Co also variations were reported. Cognitive impairment and dementia severe were associated with increased inferior uptake for patients

[34]	[^18^F]AV‐1451	The frontal, occipital, parietal, and temporal-co, as well as the amygdala, anterior and post-parahippocampus, and fusiform areas, displayed elevated levels of tau binding relative to controls in the frontal, occipital, parietal, and temporal-co, as well as the amygdala, anterior and post-parahippocampus, and fusiform sections of Alzheimer's disease and mild cognitive impairment patients

[35]	[^18^F]AV‐1451	Variation of entorhinal and neocortical tau binding was observed in patients with classic Alzheimer's disease. The tremendous memory damage being found by people with higher entorhinal and neocortical tracer retention, while those with low entorhinal and elevated neocortical attachment were the most deteriorating in other areas of neuropsychology, according to a cluster study contrasting high and low uptake groups

[36]	[^18^F]THK5317	In Alzheimer's disease patients, in addition to the midbrain, [^18^F]THK5317 binding was found in basal ganglia and thalamus. The isocratic temporal lobe and lateral parietal and frontal lobes retention were observed in the tracer retention

[37]	[^18^F]MK‐6240	In the medial temporal lobe, both amygdala, hippocampus, and parahippocampal gyrus demonstrated increased tracer uptake in patients with AS/Mild cognitive impairment. In the neocortical temporal, frontal, and parietal regions, two patients with progressive disease were taken up

[38]	[^18^F]PI‐2620	In the temporal areas, the precuneus, and the post cingulate, three Alzheimer's disease patients had asymmetric distributions of tracer retention. One Alzheimer's disease patient, who was in the early stages of the disorder, *h* Alzheimer's disease little absorption

[39]	[^18^F]RO‐948	Alzheimer's disease patients had higher tracer attachment than older controls in the right hippocampus, entorhinal area, parahippocampus, left middle-middle front lobe, fusiform gyrus, mid temporal-Co, inferior lobe, and right inferior parietal lobe

[40]	[^18^F]GTP1	Braak stage I/II brain regions have better retention of tracer in mild to moderate Alzheimer's disease patients than CN brain regions, and braak stage V/VI brain regions have higher retention of tracer

**Table 2:** Different parts of brain as feature of diagnosis.

1. Orbital frontal cortex	2. Anterior cingulate	3. Putamen
4. Prefrontal cortex	5. Posterior cingulate	6. Putamen LR
7. Superior frontal cortex	8. Occipital	9. Putamen L
10. Lateral temporal cortex	11. Global cortex	12. Putamen R
13. Medial temporal cortex	14. Amygdala	15. Putamen La
16. Posterior precuneus	17. Hippocampus	18. Putamen Lp
19. Ventral striatum	20. Caudate	21. Putamen Ra
22. Ventral striatum _LR	23. Caudate _LR	24. Putamen RP
25. Pons	26. Thalamus	27. Raphe
28. Gray matter VBM8	29. Substantia nigra	30. Raphe dorsal
31. White matter VBM8	32. Midbrain	33. Raphe nuclei
34. Brain mask GM_WM_CSF	35. Medulla	36. Centrum semiovale
37. Parietal		

**Table 3:** The SNP sequence involved.

SNP name	Sequence
rs1876152	CCGAGGTGACCTCAGGGAGGAACCAGAGAAGAAATACCCTGACTTCACTC
rs1501228	ATTAGGTAGTCAGTTCTGCACAGAAGATATGCTTCTCGTCCAAATAAATG
rs1946867	CTTCATCTTTTTTGTGTGGCAACATATGAAGCTGTACCAAATTGTATGGT

## Data Availability

For this report, the information was collected from the ADNI data set (http://adni.loni.usc.edu/).
